# Zweitschrittverfahren in der antidepressiven Pharmakotherapie: Ergebnisse aktueller Metaanalysen

**DOI:** 10.1007/s00115-024-01785-4

**Published:** 2024-12-12

**Authors:** Marlene Krabs, Tom Bschor, Jonathan Henssler, Christopher Baethge

**Affiliations:** 1 Klinik für Psychiatrie, Psychotherapie, Psychosomatik Suchtmedizin, Evang. Kliniken Essen-Mitte, Essen, Deutschland; 2 Klinik und Poliklinik für Psychiatrie und Psychotherapie, Universitätsklinikum Carl Gustav Carus an der Technischen Universität Dresden, Fetscherstraße 74, 01307 Dresden, Deutschland; 3 Regierungskommission Krankenhäuser, c/o Bundesministerium für Gesundheit, Berlin, Deutschland; 4 Klinik für Psychiatrie und Psychotherapie, Psychiatrische Universitätsklinik der Charité im St. Hedwig-Krankenhaus, Campus Charité Mitte, Berlin, Deutschland; 5 Klinik für Psychiatrie und Psychotherapie, Universität zu Köln, Köln, Deutschland

**Keywords:** Pharmakologische Depressionsbehandlung, Monotherapie, Nichtansprechen, Antidepressiva, Zweitschrittstrategie, Pharmacological depression treatment, Monotherapy, Nonresponse, Antidepressants, Second-step strategy

## Abstract

**Hintergrund:**

Antidepressive Pharmakotherapie führt häufig trotz adäquater Dauer und Dosis nicht zum gewünschten Effekt. Eine bessere Evidenz zu Zweitschrittstrategien ist notwendig.

**Ziel der Arbeit:**

Übersicht über die aktuelle Evidenzlage verschiedener pharmakologischer Zweitschrittstrategien nach dem Nichtansprechen auf eine antidepressive Monotherapie.

**Material und Methoden:**

Zusammenfassung aktueller systematischer Übersichtsarbeiten mit Metaanalysen der Autorengruppe zu pharmakologischen Zweitschrittbehandlungen.

**Ergebnisse:**

Eine Metaanalyse zeigte keinen Vorteil eines Wechsels des Antidepressivums im Vergleich zur Fortführung der bislang unwirksamen Monotherapie. Zwei Metaanalysen zeigten keinen Vorteil einer Dosiserhöhung von selektiven Serotoninwiederaufnahmehemmern (SSRI). Für selektive Serotonin- und Noradrenalinwiederaufnahmehemmer (SNRI) und trizyklische Antidepressiva (TZA) zeigten jeweils eine Metaanalyse keinen eindeutigen Vorteil einer Dosiserhöhung. Zwei Metaanalysen zeigten eine Überlegenheit einer Kombinationstherapie aus einem Wiederaufnahmehemmer (SSRI, SNRI, TZA) mit einem präsynaptischen α2-Autorezeptorantagonisten (z. B. Mirtazapin) gegenüber einer antidepressiven Monotherapie.

**Schlussfolgerungen:**

In Übereinstimmung mit den Empfehlungen der Nationalen VersorgungsLeitlinie ist bei nichtansprechender Depression die Kombination zweier Antidepressiva dem wiederholten Wechsel des Antidepressivums vorzuziehen.

## Abstract (en)

**Background:**

Antidepressant pharmacotherapy often does not result in the desired effect despite adequate duration and dose. Better evidence on second-step strategies is needed.

**Objective:**

Overview of the current evidence for various pharmacological second-step strategies after nonresponse to antidepressant monotherapy.

**Material and methods:**

Summary of recent systematic reviews with meta-analyses of the group of authors on pharmacological second-step treatment.

**Results:**

A meta-analysis showed no advantage of switching to a second antidepressant compared with continuing the previously ineffective monotherapy. Another two meta-analyses showed no benefit of increasing the dose of selective serotonin reuptake inhibitors (SSRI). For serotonin and noradrenaline reuptake inhibitors (SNRI) and tricyclic antidepressants (TCA) in each case a meta-analysis showed no clear advantage of increasing the dose. Another two meta-analyses showed a superiority of a combination therapy consisting of a reuptake inhibitor (SSRI, SNRI, TCA) with a presynaptic alpha‑2 autoreceptor antagonist (e.g., mirtazapine) compared with an antidepressant monotherapy.

**Conclusion:**

In accordance with the recommendations of the German national treatment guideline, in the event of nonresponse to antidepressant monotherapy, the combination of two antidepressants is preferable to repeated switching of the antidepressant.

Depressive Erkrankungen können grundsätzlich wirksam mit Antidepressiva behandelt werden [1]. Oftmals führt eine antidepressive Monotherapie trotz adäquater Dauer und Dosis aber nicht zum gewünschten Effekt. Metaanalysen bringen bessere Kenntnisse zur Evidenz für dann erforderliche pharmakologische Strategien des zweiten Schritts der Depressionsbehandlung.

## Hintergrund

Gemäß der Nationalen VersorgungsLeitlinie (NVL) Unipolare Depression ist eine grundsätzliche Indikation für eine antidepressive Pharmakotherapie bei einer schweren Depression und bei einer mittelgradigen Depression zumindest als gleichwertige Alternative zu einer Psychotherapie gegeben [2]. In den Zulassungs- und Wirksamkeitsstudien der verschiedenen Antidepressiva spricht ein Drittel bis die Hälfte der Patientinnen und Patienten nicht auf eine mehrwöchige Behandlung an [3, 4]. Metaanalysen, die die Effektstärke von Antidepressiva auf die depressive Symptomatik untersuchen, ergeben im Durchschnitt Werte von 0,3 [1], was nach der Konvention von Cohen [5] einem kleinen Effekt entspricht. Ein Grund für den relativ geringen Effekt könnte die Heterogenität der unter der Diagnose „Depression“ zusammengefassten Krankheitsbilder sein.

Ein relevanter Teil der Besserung während einer Antidepressivabehandlung geht auf Placebo- und unspezifische Effekte zurück. Die Arbeitsgruppe der Autoren untersuchte 2024 in einer Metaanalyse [6], die 90 randomisierte kontrollierte Studien (RCTs) einschließen konnte, wie gut sich psychische Erkrankungen unter einer reinen Placebobehandlung bessern. Es zeigte sich eine signifikante Verbesserung unter Placebo für alle neun untersuchten Erkrankungen, der Grad der Verbesserung variierte jedoch erheblich zwischen den Diagnosen. Patienten mit einer depressiven Störung erlebten die größte Verbesserung mit einer Prä-post-Effektstärke von 1,40 (95 %-Konfidenzintervall [KI] 1,24; 1,56). (Achtung: Prä-post-Effektstärken dürfen nicht mit der zuvor zitierten Effektstärke, die aus der Differenz zu einer Kontrollbedingung stammt, verglichen werden.) Dieses Ergebnis zum Verlauf depressiver Erkrankungen unter Placebo kann hilfreich sein, um die Dringlichkeit einer Antidepressivamedikation zu beurteilen und ferner, um den Krankheitsverlauf ohne spezifische Intervention zu verstehen und die Interpretation placebokontrollierter Studien zu unterstützen, bei denen der Unterschied zwischen Medikament und Placebo in der Regel das primäre Ergebnis ist.

Auch das soziale Funktionsniveau und die Lebensqualität depressiver Patientinnen und Patienten werden durch eine antidepressive Pharmakotherapie positiv, aber nur mit einem eher schwachen Effekt beeinflusst, wie zwei aktuelle Metaanalysen der Autorengruppe zeigten [7, 8].

Spricht ein Patient oder eine Patientin nicht auf einen ersten Behandlungsschritt mit einer Antidepressivamonotherapie an, sollten zunächst die Diagnose und mögliche ungünstige Einflussfaktoren auf den Therapieverlauf überprüft werden, um eine Pseudotherapieresistenz auszuschließen. Findet sich hier kein Ansatzpunkt, sollte die Behandlung verändert werden [2]. Hierfür kommen pharmakologische und nichtpharmakologische Verfahren in Betracht. Letztere, wie Psychotherapie oder Elektrokonvulsionstherapie (EKT), haben zum Teil einen hohen Stellenwert in der Depressionsbehandlung, werden in dieser Übersichtsarbeit aber nicht behandelt. Evidenzbasierte Erkenntnisse sind notwendig, um die Wirksamkeit möglicher pharmakologischer zweiter Schritte beurteilen und Therapieempfehlungen geben zu können [9].

## Methodik

Weil systematische Übersichtsarbeiten mit quantitativer (metaanalytischer) Auswertung randomisierter klinischer Studien prinzipiell den höchsten Evidenzgrad haben [10], erstellt dieser Artikel eine Übersicht über aktuelle Metaanalysen über die Wirksamkeit pharmakologischer Zweitschrittverfahren in der Depressionsbehandlung. Er konzentriert sich dabei auf Publikationen der Autorengruppe und die Empfehlungen der Nationalen VersorgungsLeitlinie Unipolare Depression (NVL; [2]).

## Ergebnisse

**Abb. 1:**
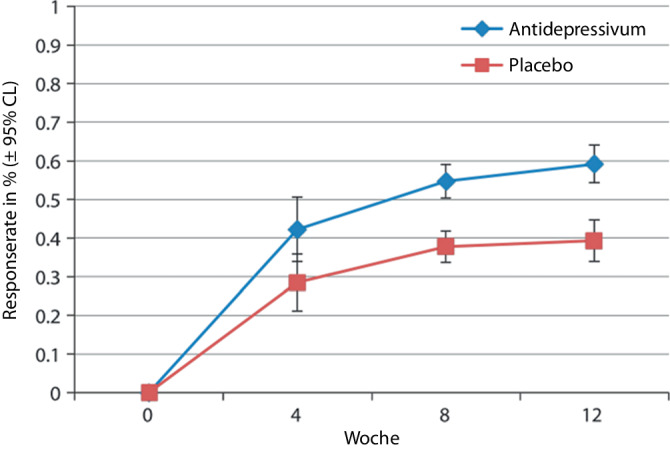
Entwicklung der Responseraten in placebokontrollierten Antidepressivastudien von mindestens 12-wöchiger Dauer. (Metaanalytische Ergebnisse nach [11], Abdruck mit freundlicher Genehmigung des Verlags. Analyse gemäß Modell zufallsbedingter Effekte [„random-effects model“].) *95* *% CL* („confidence limits“) 95 %-Konfindenzintervall

Zunächst ist eine wichtige Frage, wann überhaupt ein zweiter Schritt eingeleitet werden soll. In der aktuellen Auflage der Nationalen VersorgungsLeitlinie Unipolare Depression [2] wird empfohlen, im Falle von Nichtansprechen nach 3 bis 4 Wochen einen Strategiewechsel vorzunehmen. Bei älteren Patientinnen und Patienten soll bis zu 6 Wochen gewartet werden. Zur präziseren Einschätzung dieses Zeitraums untersuchten die Autoren 2018 in einer Metaanalyse [11] neun randomisierte kontrollierte Antidepressivastudien mit mindestens 12 Wochen Studiendauer, die kontinuierlich über Response und Remission berichteten. Die Metaanalyse zeigte, dass es bei Studienteilnehmenden, die nach 4 Wochen nicht auf ein Antidepressivum angesprochen hatten, zwischen Woche 5 und 8 noch in 22 % zu einem Ansprechen kam. Im Zeitraum von Woche 9 bis 12 erreichten dann nur noch weitere 10 % ein Ansprechen. Bei den Nonrespondern auf Placebo sprachen bis Woche 8 weitere 13 % und bis Woche 12 weitere 2,4 % an (Abb. 1).

## Zweitschrittstrategien

Bei Nonresponse auf ein Antidepressivum kann zwischen verschiedenen Zweitschrittstrategien gewählt werden. Diese werden auch in der Nationalen VersorgungsLeitlinie Unipolare Depression [2] bewertet.

### Absetzen des (wirkungslosen) Antidepressivums

Eine naheliegende, wenngleich in der Praxis eher selten angewandt Strategie ist, eine unwirksame Therapie zu beenden, im diskutierten Fall also, ein nichteffektives Antidepressivum abzusetzen. Hierbei ist das Risiko eines Absetzsyndroms zu bedenken, das sich mit vielfältigen, häufig unspezifischen somatischen und psychischen Symptomen äußern kann. Besonders häufig sind Schwindel, Übelkeit, Kopfschmerzen, Schlafstörungen und Stimmungslabilität. Absetzsymptome sind bei den meisten Patientinnen und Patienten mild und bilden sich innerhalb von 2 bis 6 Wochen zurück [12].

Nicht gut untersucht war bislang, wie häufig Absetzsymptome auftreten. Eine aktuelle Metaanalyse zu dieser Frage [13] konnte 79 Studien einschließen (44 RCTs und 35 Beobachtungsstudien), bei denen das Absetzen oder Ausschleichen eines Antidepressivums oder Placebos bei psychiatrischen Patienten untersucht wurde. Sie stellte fest, dass ungefähr jeder dritte Patient, der Antidepressiva absetzt, Absetzsymptome jeglicher Art hat (Ereignisrate 0,31; 95 %-Konfidenzintervall [KI] 0,27; 0,35). In Studien, in denen Studienteilnehmende ein Placebo absetzten, traten allerdings auch bei ungefähr einem von 6 Patienten Absetzsymptome auf (Ereignisrate 0,17; 95 %-KI 0,14; 0,21). Es zeigte sich außerdem, dass Imipramin, Paroxetin sowie Desvenlafaxin und Venlafaxin im Vergleich zu anderen Antidepressiva mit einem höheren Risiko schwerer Absetzsymptome verbunden sind.

### Wechsel des Antidepressivums

Eine systematische Übersicht aus dem Jahr 2018 [14] ist die einzige Metaanalyse, die diese in der klinischen Praxis sehr häufig angewendete Strategie untersuchte. Entscheidend zur Bewertung dieses Vorgehens ist die Wahl der geeigneten Kontrollbedingung. Die bloße Beobachtung, etwa im klinischen Alltag, dass es Patientinnen oder Patienten nach dem Wechsel eines Antidepressivums besser geht, belegt nicht, dass die Symptombesserung auf den Austausch des Antidepressivums zurückzuführen ist. Wie oben erläutert, führt bereits die reine Verlängerung der Behandlung zu einem Anstieg der Responseraten. Für die Metaanalyse wurden daher die acht RCTs identifiziert, die einen Wechsel des Antidepressivums mit der bloßen Fortführung (oder Aufdosierung) des bislang unzureichend wirksamen Antidepressivums verglichen. Es zeigte sich kein Vorteil für den Wechsel bezüglich der depressiven Symptomatik. Die Effektstärke für den Wechsel des Antidepressivums, ausgedrückt als standardisierte Mittelwertdifferenz (SMD), war mit 0,03 ([95 %-KI −0,26; 0,32], *N* = 8) praktisch null. Nach der Konvention von Cohen [5] wird erst ab einer Effektstärke von 0,2 von einem schwachen Effekt gesprochen. Auch nach Ausschluss der vier Studien, in denen eine Dosiserhöhung im Vergleichsarm erlaubt war, ergab sich kein Vorteil für den Wechsel auf ein neues Antidepressivum (SMD −0,17 [95 %-KI −0,59; 0,26], *N* = 4). Signifikante Unterschiede bei den Abbruchraten wurden nicht beobachtet.

Nach Veröffentlichung der Metaanalyse wurde eine weitere randomisierte (einfach verblindete) Studie publiziert, in der sich ein kleiner Vorteil für den Wechsel von Sertralin auf Mirtazapin gegenüber der fortgesetzten Behandlung mit dem gleichen Antidepressivum fand [15]. Die Ergänzung dieser Studie zu den vier RCTs der Metaanalyse, in denen ebenfalls keine Dosiserhöhung im Vergleichsarm erlaubt war, änderte nicht das metaanalytische Gesamtergebnis eines nicht nachzuweisenden Vorteils des Wechsels gegenüber der Fortsetzung des bisherigen Antidepressivums (SMD −0,06 [95 %-KI −0,37; 0,24], *N* = 5).

Eine Erklärung für die fehlende Wirksamkeit könnte sein, dass beinahe alle Antidepressiva zunächst ähnliche pharmakodynamische Effekte verursachen: die Erhöhung der Monoamine im synaptischen Spalt; entweder über die Wiederaufnahmehemmung (selektive Serotoninwiederaufnahmehemmer [SSRI], selektive Serotonin- und Noradrenalinwiederaufnahmehemmer [SNRI], trizyklische Antidepressiva [TZA]) oder über die Blockade der präsynaptischen Autorezeptoren (Mirtazapin, Mianserin) oder über die Hemmung der Monoaminoxidase (MAO-Hemmer). Diese primären Effekte gehen mit komplexen weiteren Einflüssen auf Stoffwechsel und Plastizität des Zentralnervensystems (ZNS), immunologische Vorgänge und oxidativem Stress einher [16], wobei noch keine integrierenden Konzepte bestehen, wie diese Veränderungen zu den klinischen Wirkungen beitragen und inwieweit sich die Antidepressiva unterscheiden.

Die Häufigkeit der Anwendung dieser Strategie steht damit im starken Gegensatz zu ihrer geringen und negativen wissenschaftlichen Evidenz. Die NVL Unipolare Depression [2] beurteilt daher diese Option als den anderen Strategien bei Nichtansprechen nachgeordnet. Maximal ein einmaliger Wechsel wird als vertretbar eingeschätzt, nicht jedoch ein sukzessives Ausprobieren mehrerer Antidepressiva. Aufgrund der Unsicherheit formuliert die Leitlinie eine „Kann“-Empfehlung. Die Empfehlung bezieht sich dabei nicht auf einen Antidepressivawechsel, der aufgrund von Unverträglichkeit des Medikaments durchgeführt wird.

### Dosiserhöhung des Antidepressivums

#### SSRI

Bereits 2005 kam eine systematische Übersichtsarbeit zu dem Ergebnis, dass für SSRI keine positive Dosis-Wirkungs-Beziehung besteht und eine Dosiserhöhung aufgrund einer erhöhten Rate an Nebenwirkungen eventuell sogar nachteilig ist [17]. Eine große Metaanalyse bestätigte einen sehr geringen Zusammenhang von Ansprechen auf eine SSRI-Behandlung und SSRI-Dosis [18].

Aktuelle Untersuchungen bestätigen dieses Ergebnis: Eine systematische Metaanalyse von 2018 [14] schloss neun RCTs ein, bei denen zuvor eine SSRI-Behandlung mit Standarddosierung unzureichend war, und konnten keinen Vorteil für eine Dosiserhöhung gegenüber der Fortführung der Standarddosis finden (SMD 0,05 [95 %-KI −0,14; 0,25], *N* = 9). Die Autoren untersuchten 2020 in einer weiteren Metaanalyse [16] randomisierte Studien mit unterschiedlichen SSRI-Dosierungen mit einer Behandlungsdauer von mindestens 3 Wochen. Sie konnten 33 RCTs einschließen (deutlich mehr als in die Metaanalyse von 2018, da im Vergleich dazu die Vorbedingung der gescheiterten SSRI-Medikation wegfiel). Die Vergleiche von Dosierungsgruppen (niedrig, mittel und hoch) zeigten keine signifikanten Unterschiede; am stärksten noch, aber ebenfalls unter der Schwelle für eine klinische Relevanz, bezogen auf mittlere vs. niedrige Dosen (SMD −0,15 [95 %-KI −0,28; −0,01]). Eine höhere Dosis von SSRI führte aber zu mehr Studienabbrüchen. Bei der Betrachtung der einzelnen SSRIs ergab sich für Fluoxetin der interessante Befund, dass die hohe Dosierung von 60 mg am Tag signifikant schlechter antidepressiv wirksam war als die niedrige Standarddosis von 20 mg (SMD 0,2 [95 %-KI 0,01; 0,39]).

#### SNRI

Eine weitere systematische Übersichtsarbeit [16] verglich 2022 unterschiedliche Dosierungen von SNRI. Die Metaanalyse fand keine klinisch oder statistisch signifikanten Unterschiede zwischen niedrigen, mittleren und hohen Dosierungen (hoch vs. mittel: SMD −0,06 [95 %-KI −0,16; 0,03], *N* = 29), auch nicht in der Analyse der einzelnen Substanzen einschließlich Venlafaxin. Die Annahme, dass es erst bei höheren Venlafaxindosierungen zu einer relevanten Noradrenalinwiederaufnahmehemmung und dadurch zu einem zusätzlichen antidepressiven Effekt kommt, ließ sich, in Übereinstimmung mit anderen Metaanalysen [18], klinisch also nicht zeigen. Eine höhere Dosis des SNRI führte aber zu mehr Studienabbrüchen aufgrund von Nebenwirkungen [21]. Wie bei SSRI sprechen die Ergebnisse nicht für eine Dosis-Wirkungs-Beziehung innerhalb des therapeutisch angewandten Dosisbereichs.

#### Trizyklische Antidepressiva

Eine systematische Übersichtsarbeit von RCTs [22] untersuchte 2022 Dosisvergleichsstudien mit trizyklischen Antidepressiva. Sie schloss 15 randomisierte Studien mit unterschiedlichen Dosierungen ein. Die standardisierte Mittelwertdifferenz (SMD) lag bei 0,34 ([95 %-KI −0,03; 0,70], *N* = 15) für eine Dosiserhöhung um 100 mg/Tag, war aber nicht statistisch signifikant (*p* = 0,073). Während mehrere Einzelvergleiche keinen Dosisunterschied zeigten, waren 300 mg Imipramin/Desipramin 150 mg/Tag statistisch signifikant überlegen (SMD 0,80 [95 %-KI 0,28; 1,33], *p* = 0,003). Die Zahl der Studienabbrecher nahm mit höheren Dosen zu, wenn auch statistisch nicht signifikant.

**Abb. 2:**
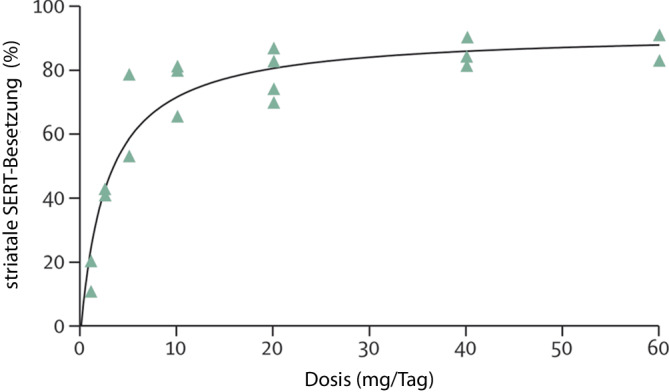
Hyperbolische Beziehung zwischen Tagesdosis Citalopram und Besetzung des Serotonintransporters (*SERT*) im Striatum ([24], Abdruck mit freundlicher Genehmigung des Verlages)

Das Fehlen einer Dosis-Wirkungs-Beziehung zumindest für SSRI und SNRI passt gut zur bekannten Beziehung von Dosis und Pharmakodynamik. Die Medikamente beider Gruppen blockieren im Gehirn die Serotoninwiederaufnahmetransporter. In Studien mit funktioneller Bildgebung konnte gezeigt werden, dass das Ausmaß der Blockade der Wiederaufnahmetransporter in einer hyperbolischen Beziehung zur Dosis der Pharmaka steht [23]. Beispielhaft wird dies in Abb. 2 für Citalopram illustriert [24, 25]: Bereits bei einer niedrigen Standarddosierung von 20 mg/Tag sind ca. 80 % der Serotoninwiederaufnahmetransporter blockiert. Bei Verdoppelung bzw. Verdreifachung der Dosis steigt die Blockade lediglich auf ca. 85 % bzw. 88 %. Selbst bei 10 mg am Tag sind noch ca. 76 % der Wiederaufnahmetransporter blockiert.

Die NVL Unipolare Depression [2] rät von der Strategie einer Dosiserhöhung bei SSRI ab, da eine Dosis-Wirkungs-Beziehung für SSRI nicht gefunden werden konnte (moderate Evidenzqualität). Für eine Dosiserhöhung bei Nicht-SSRI ist die Evidenzqualität sehr niedrig. Den fehlenden Belegen für mögliche Vorteile steht eine zu erwartende höhere Rate unerwünschter Arzneimittelwirkungen gegenüber, sodass die Leitlinie ebenfalls davon abrät, die Dosis von Nicht-SSRI bei Nichtansprechen über die Standarddosierung oder den empfohlenen Serumspiegel hinaus zu erhöhen, wobei der Empfehlungsgrad aufgrund der geringen Evidenzbasis für diese Substanzen abgeschwächt wurde. Die Empfehlungen gelten nicht, wenn ein Medikamentenserumspiegel unterhalb des therapeutischen Bereichs gemessen wurde.

### Kombination zweier Antidepressiva

Die Kombination zweier Antidepressiva ist eine weitere potenzielle Zweitschrittstrategie, wenn ein Antidepressivum allein trotz adäquater Dosis und Dauer nicht zum gewünschten Behandlungserfolg führte. Nur im Vergleich zu einer Antidepressivamonotherapie lässt sich beurteilen, ob eine mögliche Symptombesserung tatsächlich auf die Addition eines zweiten Antidepressivums zurückzuführen ist. 2016 wurde eine erste Metaanalyse der Arbeitsgruppe zur Kombinationsbehandlung zweier Antidepressiva [26] veröffentlicht, welche 38 Studien einschloss, darunter sechs nichtrandomisierte. Es zeigte sich eine signifikante Überlegenheit der Kombinationstherapie über die Monotherapie (SMD 0,29 [95 %-KI 0,16; 0,42]). Bereits in dieser ersten Metaanalyse ging die positive Wirkung ausschließlich auf einen bestimmten Kombinationstyp zurück (Wiederaufnahmehemmer mit Autorezeptorblocker; siehe unten), für den der Effekt entsprechend deutlicher ausfiel (SMD 0,54 [95 %-KI 0,29; 0,79]). Im Einklang mit den Befunden zur Dosiserhöhung von Antidepressiva zeigte sich zudem in einer Analyse auf Basis von Imipraminäquivalenzdosen, dass die bessere Wirkung der Kombinationen keineswegs nur auf eine erhöhte Antidepressivagesamtdosis zurückzuführen war. Vielmehr ergeben sich bei der Kombinationstherapie offenbar pharmakodynamische und klinische Synergismen.

Eine aktuellere Metaanalyse von 2022 [27] konnte 39 RCTs einschließen. Eine Kombinationsbehandlung erwies sich auch in dieser Analyse als statistisch signifikant überlegen gegenüber einer Monotherapie (SMD 0,31 [95 %-KI 0,19; 0,44]). Auch hier war speziell die Kombination eines Wiederaufnahmehemmers (SSRI, SNRI oder trizyklisches Antidepressivum) einerseits mit einem präsynaptischen α2-Autorezeptorantagonisten andererseits (Mianserin, Mirtazapin, Trazodon) effektiv (SMD 0,37 [95 %-KI 0,19; 0,55], *N* = 18). Dies galt auch in den reinen Nonresponderpopulationen (SMD 0,24 [95 %-KI 0,03; 0,45], *N* = 12) sowie bei Erstbehandlung (SMD 0,64 [95 %-KI 0,12; 1,15], *N* = 5).

Die spezifische Wirksamkeit dieses Kombinationstyps ist in Übereinstimmung mit theoretischen pharmakologischen Überlegungen, weshalb diese Strategie recht gut untersucht ist. Durch die Hemmung der Monoaminwiederaufnahme mit einem SSRI, einem SNRI oder einem TZA kommt es erwünschterweise zu einer Erhöhung der Serotonin- und/oder Noradrenalinkonzentration im synaptischen Spalt. Hierdurch werden aber auch die präsynaptischen α2-Autorezeptoren stimuliert. Die α2-Autorezeptoren regulieren physiologischerweise die Konzentration der Neurotransmitter im synaptischen Spalt, indem sie bei hohen Konzentrationen durch negative Rückkoppelung den weiteren Anstieg der Serotonin- und/oder Noradrenalinkonzentration abbremsen. Bei der alleinigen Gabe eines Wiederaufnahmehemmers kommt es so zu einer unerwünschten Abschwächung der pharmakologischen Wirkung über die Stimulation der α2-Autorezeptoren. Durch die Kombination mit einem α2-Autorezeptorantagonisten wird nach dieser Theorie die unerwünschte Abschwächung verhindert. Darüber hinaus ergeben sich bei dieser Kombination spezifische klinische Synergismen – so können die eher sedierenden α2-Autorezeptorantagonisten die mit Monoaminwiederaufnahmehemmern assoziierte Unruhe, Agitation und sexuelle Dysfunktion ausgleichen.

Die naheliegende Kombination serotonerger oder serotonerg-noradrenerger Antidepressiva mit dem dopaminergen Bupropion hingegen war der Monotherapie nicht überlegen (SMD 0,10 [95 %-KI −0,07; 0,27], 7 RCTs), sodass es keine positive Evidenz für die Empfehlung dieser Kombination gibt.

In beiden Metaanalysen erwies sich die Kombinationsbehandlung als gut verträglich. Die Zahl der vorzeitigen Studienabbrüche aufgrund unerwünschter Arzneimittelwirkungen war bei den kombiniert behandelten Studienteilnehmern nicht höher als bei den monotherapeutisch behandelten.

Die Autoren schlussfolgerten, dass die Kombinationstherapie von SSRI, SNRI oder TZA einerseits mit einem präsynaptischen α2-Autorezeptorenantagonisten (etwa Mianserin oder Mirtazapin) andererseits eine wirksame und sichere Behandlungsoption für Patientinnen und Patienten sein kann, die auf eine antidepressive Monotherapie nicht ansprechen. Bei Mianserin ist das Risiko einer Agranulozytose zu beachten.

Die Leitlinie empfiehlt spezifisch den oben genannten Kombinationstyp und bewertet dieses Vorgehen als eine Strategie der 1. Wahl bei Nichtansprechen einer Antidepressivamonotherapie (Empfehlung „sollte“). Eine „Sollte“-Empfehlung vergibt die NVL ferner für eine Augmentation mit Lithium oder atypischen Neuroleptika (zugelassen: Quetiapin, off-label: Aripiprazol, Risperidon oder Olanzapin), für die jeweils ältere systematische Metaanalysen ebenfalls eine Wirksamkeit gegenüber einer Placeboaugmentation zeigten [28–30].

Als pharmakologisches Vorgehen nach Nonresponse – genauer gesagt, nach dem Versagen von mindestens zwei Antidepressiva – ist ferner die Addition von Esketaminnasenspray zum Antidepressivum zugelassen. Die Wirksamkeit gegenüber einem Placebonasenspray konnte metaanalytisch mehrfach gezeigt werden [31–33]. Die NVL [2] spricht eine „Kann“-Empfehlung für mittelgradige und schwere depressive Episoden nach mehreren erfolglosen Behandlungsversuchen aus. Für Psilocybin wurden in kleineren Phase-II-Studien zum Teil eindrucksvolle antidepressive Effekte gefunden [34], allerdings bezog sich die Mehrzahl der Studien nicht auf Teilnehmende mit zuvor gescheiterter Antidepressivabehandlung, und Psilocybin ist bisher eine verbotene Substanz, die nicht für den Behandlungsalltag zur Verfügung steht.

Eine Übersicht über die aktuelle Evidenz dieser Zweitschrittstrategien zeigt Tab. 1.

**Tab. 1:** Übersicht zur Evidenz von Zweischrittstrategien der antidepressiven Pharmakotherapie

Zweitschrittstrategie	Metaanalyse		Empfehlung NVL [2] (Stand 2022)
	Autoren und Jahr	Ergebnis	
*Wechsel des AD*	Bschor et al. 2018 [14]	In 4 bzw. 8 RCTs kein Vorteil für Wechsel gegenüber Fortführung des bislang unwirksamen Antidepressivums	Maximal einmaliger Wechsel vertretbar; „Kann“-Empfehlung
*Aufdosieren des AD*	Rink et al. 2018 [19]	9 RCTs mit SSRI: kein signifikanter besserer Effekt einer höheren gegenüber einer niedrigeren Dosis	SSRI: starke Negativempfehlung „soll nicht“ MAO-Inhibitoren, SNRI, TZA: abgeschwächte Negativempfehlung „sollte nicht“
	Braun et al. 2020 [20]	33 RCTs: keine positive oder konsistente Evidenz für eine Dosis-Wirkungs-Beziehung bei SSRI	
	Rink et al. 2022 [21]	26 RCTs: kein signifikanter Effekt einer höheren Dosis von SNRI gegenüber einer Standarddosierung	
	Baethge et al. 2022 [22]	15 RCTs: nichtsignifikanter positiver Effekt einer Dosiserhöhung von TZA; 300 mg besser als 150 mg/Tag	
*Kombination zweier AD*	Henssler et al. 2022 [27]	39 RCTs: signifikanter Vorteil einer Kombinationsbehandlung aus einem Wiederaufnahmehemmer (SSRI, SNRI oder TZA) einerseits mit einem präsynaptischen α2-Autorezeptorantagonisten andererseits (Mianserin, Mirtazapin, Trazodon) im Vergleich zur Monotherapie	SSRI, SNRI oder TZA + präsynaptischer α2-Autorezeptorantagonist: abgeschwächte positive Empfehlung – „sollte“

*AD* Antidepressivum, *MAO* Monoaminoxidase, *NVL* Nationale VersorgungsLeitlinie, *RCT *randomisierte kontrollierte Studie, *SSRI* selektive Serotoninwiederaufnahmehemmer, *SNRI* selektive Serotonin- und Noradrenalinwiederaufnahmehemmer, *TZA* trizyklische Antidepressiva

## Fazit für die Praxis

- Eine antidepressive Pharmakotherapie führt im ersten Schritt oftmals nicht zum gewünschten Erfolg, Zweitschrittbehandlungen sind häufig notwendig.
- Aus den hier referierten Metaanalysen lässt sich keine wissenschaftliche Evidenz für die Wirksamkeit eines Wechsels des Antidepressivums als Zweitschrittbehandlung ableiten.
- Für SSRI konnte keine Dosis-Wirkungs-Beziehung bestätigt werden.
- Auch für SNRI gibt es keine Bestätigung einer Dosis-Wirkungs-Beziehung.
- Die klinische Evidenz spricht für eine Kombination von SSRI, SNRI oder trizyklischen Antidepressiva einerseits mit Mianserin, Mirtazapin oder Trazodon andererseits als wirksame Zweitschrittbehandlung.
- Auch die Lithium‑, Quetiapin- und Esketaminaugmentation sind evidenzbelegte Zweitschrittstrategien.
- Nur für schwere Depressionen besteht eine generelle Empfehlung für den Beginn einer pharmakologischen Behandlung. Inwieweit dies auch für weitere Behandlungsschritte nach Nonresponse gilt, ist unklar. Die Beendigung einer wirkungslosen Pharmakotherapie kann ein rationales Vorgehen sein.
