# Erratum zu: Zweitschrittverfahren in der antidepressiven Pharmakotherapie

**DOI:** 10.1007/s00115-025-01827-5

**Published:** 2025-04-03

**Authors:** Marlene Krabs, Tom Bschor, Jonathan Henssler, Christopher Baethge

**Affiliations:** 1 Klinik für Psychiatrie, Psychotherapie, Psychosomatik Suchtmedizin, Evang. Kliniken Essen-Mitte, Essen, Deutschland; 2 Klinik und Poliklinik für Psychiatrie und Psychotherapie, Universitätsklinikum Carl Gustav Carus an der Technischen Universität Dresden, Fetscherstraße 74, 01307 Dresden, Deutschland; 3 Regierungskommission Krankenhäuser, c/o Bundesministerium für Gesundheit, Berlin, Deutschland; 4 Klinik für Psychiatrie und Psychotherapie, Psychiatrische Universitätsklinik der Charité im St. Hedwig-Krankenhaus, Campus Charité Mitte, Berlin, Deutschland; 5 Klinik für Psychiatrie und Psychotherapie, Universität zu Köln, Köln, Deutschland

**Erratum zu:**

**Nervenarzt 2025**

10.1007/s00115-024-01785-4

Der Artikel *Zweitschrittverfahren in der antidepressiven Pharmakotherapie* von Marlene Krabs et al. wurde ursprünglich Online First ohne Open Access auf der Internetplattform des Verlags publiziert. Nach der Veröffentlichung in Bd. 96, Heft 2, pp. 138–145 hatten sich die Autoren für eine Open-Access-Veröffentlichung entschieden. Das Urheberrecht des Artikels wurde deshalb in © Der/die Autor(en) 2025 geändert. Dieser Artikel ist jetzt unter der Creative-Commons-Namensnennung 4.0 International-Lizenz veröffentlicht, welche die Nutzung, Vervielfältigung, Bearbeitung, Verbreitung und Wiedergabe in jeglichem Medium und Format erlaubt, sofern Sie den/die ursprünglichen Autor(en) und die Quelle ordnungsgemäß nennen, einen Link zur Creative-Commons-Lizenz beifügen und angeben, ob Änderungen vorgenommen wurden.

Die in diesem Artikel enthaltenen Bilder und sonstiges Drittmaterial unterliegen ebenfalls der genannten Creative-Commons-Lizenz, sofern sich aus der Abbildungslegende nichts anderes ergibt. Sofern das betreffende Material nicht unter der genannten Creative-Commons-Lizenz steht und die betreffende Handlung nicht nach gesetzlichen Vorschriften erlaubt ist, ist für die oben aufgeführten Weiterverwendungen des Materials die Einwilligung des jeweiligen Rechteinhabers einzuholen.

Weitere Details zur Lizenz entnehmen Sie bitte der Lizenzinformation auf http://creativecommons.org/licenses/by/4.0/deed.de.

